# *Vogesella urethralis*-induced aspiration pneumonia and bacteremia in an elderly man: a first case report and literature review

**DOI:** 10.1186/s12879-023-08269-x

**Published:** 2023-05-04

**Authors:** Hironari Matsuda, Isana Katayama, Takayasu Watanabe, Arisa Komatsu, Naoko Iwakami, Yukiko Goto, Misako Suzuki, Shin-ichiro Iwakami, Kazuhisa Takahashi

**Affiliations:** 1 Department of Respiratory Medicine, Juntendo University School of Medicine Graduate School of Medicine, 3-1-3 Hongo, Bunkyo-Ku, Tokyo 113-8431Tokyo Japan; 2 Department of Respiratory Medicine, Juntendo Shizuoka Hospital, Izunokuni, Shizuoka Japan; 3 Department of Clinical Laboratory Medicine, Juntendo Shizuoka Hospital, Izunokuni, Shizuoka Japan

**Keywords:** *Vogesella urethralis*, *Vogesella* species, 16S rRNA gene sequence analysis, Pneumonia, Bacteremia

## Abstract

**Background:**

*Vogesella* species are common aquatic Gram-negative rods that were first reported in 1997. *Vogesella urethralis* bacterium was first isolated from human urine in 2020. Only two cases of disease caused by *Vogesella* species have been reported with no case of *Vogesella urethralis*-caused disease being reported as yet. Herein, we report a case of aspiration pneumonia and bacteremia caused by *Vogesella urethralis*.

**Case presentation:**

An 82-year-old male patient was admitted with dyspnea, increased sputum production, and hypoxia. Gram-negative rods were isolated from the blood and sputum cultures of the patient. He was diagnosed with aspiration pneumonia and bacteremia. Initially, *Vogesella urethralis* was wrongly identified as *Comamonas testosteroni* based on fully automated susceptibility testing; however, additional 16S rRNA gene sequencing identified the causative as *Vogesella urethralis*. The patient was treated with piperacillin and tazobactam. Unfortunately, he developed aspiration pneumonia again and died during hospitalization.

**Conclusions:**

Since no database exists for rare bacteria in traditional clinical microbiology laboratories, 16S rRNA gene sequence analysis is useful. We report the first case of *Vogesella urethralis*-induced aspiration pneumonia and bacteremia.

**Supplementary Information:**

The online version contains supplementary material available at 10.1186/s12879-023-08269-x.

## Background

The *Vogesella* species belongs to the *Neisseriaceae* family (order, Neisseriales; class, Betaproteobacteriaceae) and is a Gram-negative, rod-shaped, aerobic, or chemoheterotrophic bacteria. *Vogesella* was first described in 1997 by Grimes et al. [1]. Several species of *Vogesella* have been subsequently discovered. *Vogesella urethralis* isolated from human urine was first reported by Yu et al. in 2020 [2]. It is a Gram-negative, rod-shaped, aerobic, motile, non-spore-forming, and poly-β-hydroxybutyrate-accumulating bacterium and is positive for oxidase, catalase, and DNase. These bacteria can also hydrolyze gelatin, casein, and tween 20, 40, 60, and 80 but not aeslin, starch, chitin, and carboxymethyl-cellulose. This is the first case report of *Vogesella urethralis*-induced aspiration pneumonia and bacteremia.

## Case presentation

**Fig. 1:**
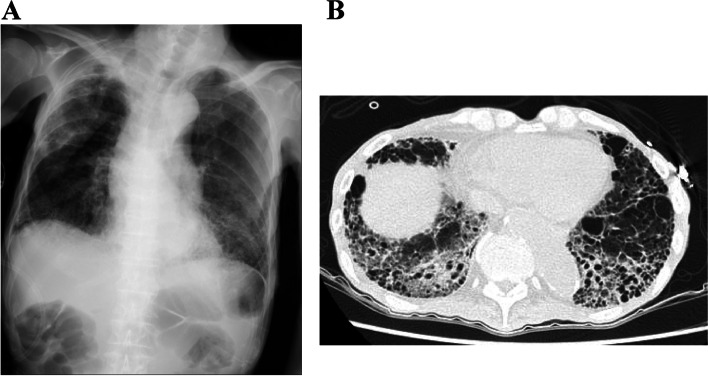
Chest radiographs and Thoracic computed tomography. **a**: Chest radiographs show frosted shadows in the bilateral lower lung fields. **b**: Thoracic computed tomography shows diffuse emphysematous changes with new infiltrative and ground-glass opacity in the bilateral lung bases

An 82-year-old male patient was under observation for stage 5 chronic renal failure, emphysema, and chronic pulmonary aspergillosis. He had been a smoker (39 pack-years). He was on some medications but not immunosuppressive agents, including inhaled/oral corticosteroids. Four days before his admission to our hospital, he had dyspnea on exertion and increased sputum production. When he arrived at our hospital, he was conscious, with a blood pressure, pulse rate, percutaneous arterial oxygen saturation, respiratory rate, and body temperature of 112/56 mmHg, 86 bpm (with a regular rhythm), 77% (on room air), 18 breaths/min, and 36.4 ℃, respectively. The results of blood tests (sample collected in the emergency room) were as follows: white blood cell count, 21.0 × 10^9^/L; neutrophil percentage, 90.7%; absolute neutrophil count, 19.0 × 10^9^/L; C-reactive protein, 31.9 mg/dL; presepsin, 1,793 pg/mL; albumin, 2.6 g/dL; blood urea nitrogen, 75.9 mg/dL; and creatinine, 3.05 mg/dL. Chest radiographs showed frosted shadows in the bilateral lower lung fields. A thoracic computed tomography scan indicated diffuse emphysematous changes with new infiltrative and ground-glass opacities in the bilateral lung bases (Fig. 1). The patient was admitted to the hospital and treated with ampicillin and sulbactam for aspiration pneumonia. However, gram-negative bacilli were detected in blood cultures on the second day of admission. Therefore, we changed the antibiotics to piperacillin and tazobactam. Aspiration pneumonia developed again on the 15th day of hospitalization. Unfortunately, he passed away on the 18th day.

**Fig. 2:**
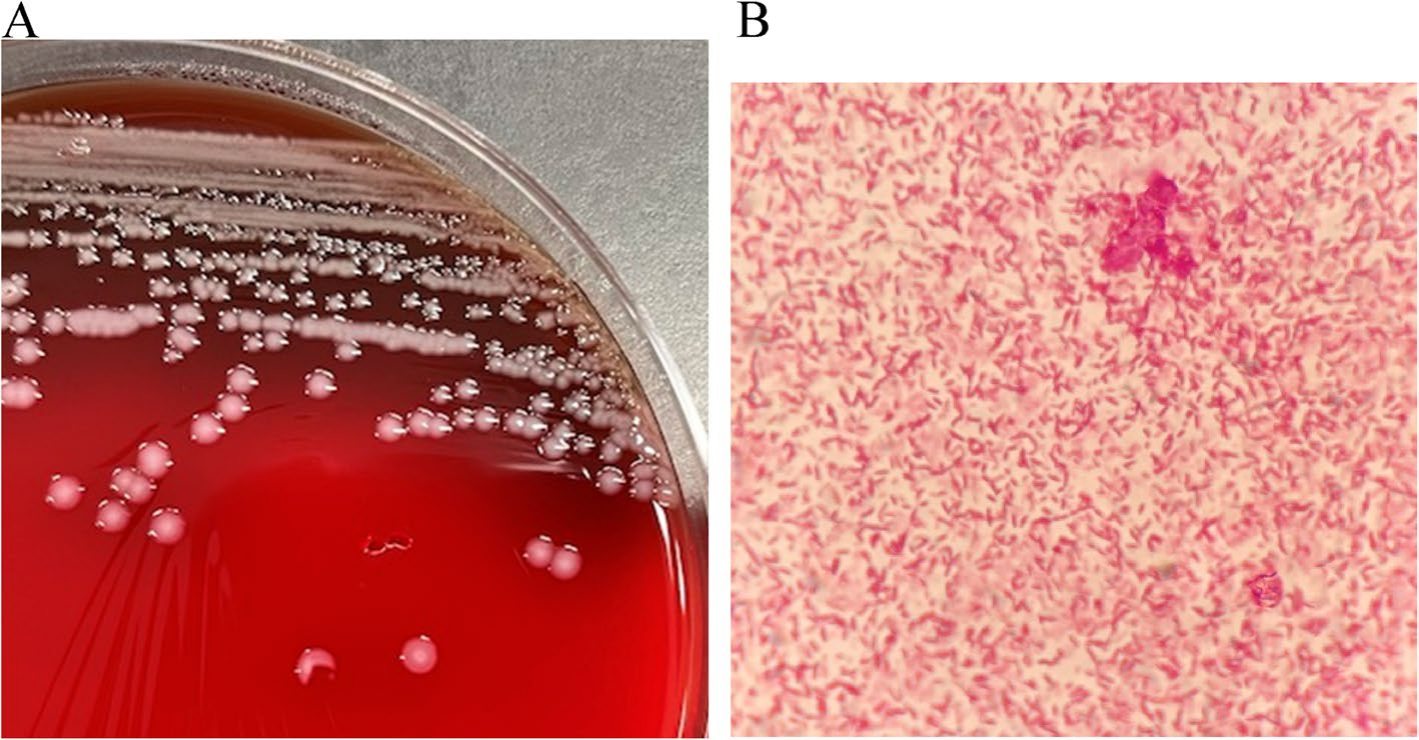
*Vogesella urethralis* identification. **a**: Strains isolated on blood agar. The colonies formed are visible, round, entirely convex, and white. **b**: Gram-negative, aerobic, spore-free, rod-shaped bacteria. Total magnification, 1000x

Blood, sputum, and urine were collected for culture at the time of admission. The sputum smear test showed gram-negative rods with white blood cells. BD PhoenixTM M 50 (Becton, Dickinson, Japan) identified two types of gram-negative rods from the sputum culture; one was *Klebsiella oxytoca* and the other was unnamed. Moreover, the latter bacteria were abundant. The urine culture was negative. Two sets of blood cultures were performed on the BACTEC FX (Becton, Dickinson, Japan) automated blood culture analyzer using 23F aerobic and 22F anaerobic raisin bottles. One aerobic bottle was positive within 14 h. Gram-staining with Fiber G (Nihon Pharmaceutical Co., Ltd., Japan) showed elongated Gram-negative rods suspected to be the same as the bacteria from the sputum (Fig. 2). Furthermore, growth on Trypticase Soy Agar II with 5% sheep blood on Drigalski Agar (Becton, Dickinson, Japan) at 35 ℃ for 24 h in both sputum and blood specimen was seen. Initially, BD PhoenixTM M 50 was used to analyze the sample, and it identified a rare bacterium, *Comamonas testosteroni*. Therefore, species identification was conducted again using API 20NE (BioMérieu); however, it was impossible to make an appropriate identification. Subsequently, 16S rRNA gene sequence analysis was performed and revealed similar isolates as *Vogesella urethralis* (99.7%, GenBank Accession Number NR_169490.1), *Vogesella perlcida* (99.36%, GenBank Accession Number NR_044326.1), and *Vogesella amnigena* (99.56%, GenBank Accession Number NR_137334.1) (Table 1). The drug susceptibility of the organism was determined by the disc method using Brucella Hemin and VitaminK1 agar (Kyokuto Pharmaceutical Co., Ltd., Tokyo, Japan). The organism was found to be susceptible as it showed a blocking circle of more than 30 mm for all drugs except fosfomycin (Table 2). The susceptibility results were interpreted with reference to *Neisseriaceca* species as listed in CLSI M100-S27. Therefore, based on the 16S rRNA gene sequence analysis and culture reports, we diagnosed *Vogesella urethalis*-induced bacteremia.

**Table 1:** 16S rRNA gene sequence analysis results

Results	Sequence similarity	GenBank Accession No
Vogesella urethralis	1419/1423 (99.72%)	NR_169490.1
Vogesella perlucida	1388/1397 (99.36%)	NR_044326.1
Vogesella amnigena	1373/1393 (99.56%)	NR_137334.1

**Table 2:** Susceptibility results by disk methods

Antibiotic	Antibiogram result	Zone Diameter
Piperacillin	Susceptible	≧ 30 mm
Cefazolin	Susceptible	≧ 30 mm
Ceftriaxone	Susceptible	≧ 30 mm
Ceftazidime	Susceptible	≧ 30 mm
Cefepime	Susceptible	≧ 30 mm
Flomoxef	Susceptible	≧ 30 mm
Imipenem / Cilastatin	Susceptible	≧ 30 mm
Aztreonam	Susceptible	≧ 30 mm
Gentamicin	Susceptible	≧ 30 mm
Amikacin	Susceptible	≧ 30 mm
Minocycline	Susceptible	≧ 30 mm
Fosfomycin	Resistant	0 mm
Levofloxacin	Susceptible	≧ 30 mm

## Discussion and conclusions

We report a case of pneumonia and bacteremia caused by *Vogesella urethralis.* The genus *Vogesella* was originally called *Pseudomonas indigofera* and was renamed *Vogesella indigofera* in 1997 [1]. *V. indigofera* has been isolated from oxidation pond sediments, whereas others have been found in hot springs, river water, and the rice rhizosphere [2]. Since then, several *Vogesella* species have been reported.

We reviewed a few reports in which *Vogesella* spp was isolated from human specimens (Table 3). This review was conducted by searching PubMed and ICHUSHI Web from their inception to January 14, 2023, using the term, “*Vogesella*”. Reference lists from sources were manually searched for additional references. *V. urethralis* was reported by Lan et al. for the first time. However, the pathogenicity was unknown [3], and only*Vogesella perlucida* has been reported as a human pathogen in the genus *Vogesella* [2] [4].

**Table 3:** Characteristics of patients from whom Vogesella species were isolated

	Age	Sex	Source of the infection	Symptoms	History	Immunosuppressive agent	Specimen	Bacteria	Identification methods	Diagnosis	Susceptibility	Used antibiotics	Mortality
Yu et al., 2020, China [2]	71	female	river snail	fever, inflamed limbs	chronic heart failure, chronic bronchitis, abnormal renal function, rheumatoid arthritis	dexamethasone	blood	*V. perlucida*	16 s rRNA gene analysis	bacteremia, soft tissue infection	described in Table S 1	Vancomycin, Levofloxacin	alive
Kuroiwa et al., 2020, Japan [4]	46	female	hot spring	fever, low back pain	fibroid, endometriosis	not described	blood	*V. perlucida*	16 s rRNA gene analysis	bacteremia, pyosalpinx	not described	Ceftriaxone, Ampicillin/sulbactam	alive
Lan et al., 2020, China [3]	not described	not described	not described	not described	chronic renal, failure	not described	urine	*V.urethralis*	16 s rRNA gene analysis	not described	described in Table S 2	not described	not described
Our case, Japan	82	male	unknown	fever, sputum, dyspnea	chronic renal failure, emphysema, chronic pulmonary aspergillosis	none	blood, sputum	*V.urethralis*	16 s rRNA gene analysis	bacteremia, pneumonia	described in Table 2	Ampicillin/sulbactam, Piperacillin/tazobactam	died

Bacteremia was observed in three cases, including ours. Moreover, chronic renal failure was observed as the underlying disease in most cases. The two cases caused by *V. perlucida* responded well to antimicrobial therapy.

In our case, an 82-year-old male patient presented with dyspnea. He also had chronic renal failure. This is the first report of a case of *Vogesella* pneumoniae infection. Initial treatment was effective, but pneumonia recurred, and the patient passed away. Chronic renal failure may be a risk factor for developing the disease.

Although *V. perlucida* was first reported to have been isolated from spring water. Snails and hot springs are thought to be the routes of infection for the two cases as well. On the other hand, the route of infection of *V. urethralis* is unknown. The route of infection may be useful in the differential diagnosis. *Vogesella* species are assumed to cause opportunistic infections.

Three cases were misidentified as *Sphingomonas paucimobilis* by the Vitek2system (BioMérieux Inc., France), and our case was misidentified as *Comamonas testosteroni* by BD PhoenixTM M 50. In the case of Yu et al., they tried the MALDI-TOF analyzer but failed to detect the causative pathogen [2]. In our case, our lab technicians used an API 20NE to support the result from BD PhoenixTM M 50, but we also failed. Finally, these researchers and our team confirmed the pathogen by 16 s rRNA gene analysis.

16 s rRNA gene analysis is a comprehensive method to identify bacteria by PCR targeting 16 s rRNA genes and comparing them with a database. The 16 s rRNA analyses are reported with homology results from public databases and molecular phylogenetic tree analysis. rRNA is RNA that makes up the ribosome, and in bacteria, it is classified into 23S rRNA, 16S rRNA, and 5S rRNA. Woose et al. proposed a statistical classification method for all organisms using small subunits rRNA gene sequences [5], and 16 s rRNA sequences are used for the statistical classification of bacteria. There is no consensus on the boundaries of genetic differences for species identification, but homology in the range of 99–99.5% is often used in practice. However, if a single nucleotide is different, it should be considered a separate species. In the Dendrogram, the absence of lateral branches between known species indicates the same molecular position as the specimen. On the other hand, when lateral branches are present, identification at the species level is difficult; detection at the genus level is required. In this case, the isolate from the blood was 99.72% homologous to *V. urethralis*, and no lateral branches were depicted in the Dendrogram. Finally, we identified the strain isolated from the blood and sputum as *Vogesella urethralis* and reported a rare case of *Vogesella urethralis*-induced aspiration pneumonia and bacteremia.

Automatic identification instruments, including the BD PhoenixTM M 50, API 20NE, and Vitek-2 system, might be considered insufficient for accurate identification. Therefore, further 16S rRNA gene sequencing is useful when rare Gram-negative rods are identified by automated instruments.

## Supplementary Information

**Additional file 1: Figure S1.** Dendrogram based on 16S rRNA gene sequences of the specimen from bloodand its closely related species. Bar 0.02 substitution per nucleotide position.

**Additional file 2: Table S1.** Susceptibility results of a case described by Yu et al., 2020.

**Additional file 3: Table S2.** Susceptibility results of a case described by Lan et al., 2020.

## Data Availability

The datasets used and/or analyzed during the current study are available from the corresponding author upon reasonable request. Also, the datasets generated and/or analyzed during the current study are available in the DNA Data Bank of Japan repository, DRA015402.
