# Supplementary material for: Vogesella urethralis-induced aspiration pneumonia and bacteremia in an elderly man: a first case report and literature review
Source: BMC Infect Dis. 2023 May 4;23:285. doi: 10.1186/s12879-023-08269-x (PMC10157996; doi:10.1186/s12879-023-08269-x)
Supplement: Supplementary file 1 — Additional file 1: Figure S1. Dendrogram based on 16S rRNA gene sequences of the specimen from bloodand its closely related species. Bar 0.02 substitution per nucleotide position. [file 12879_2023_8269_MOESM1_ESM.docx]

Figure S1. Dendrogram based on 16S rRNA gene sequences of the specimen from blood (labeled as unknown) and its closely related species. Bar 0.02 substitution per nucleotide position.
