# Supplementary material for: Vogesella urethralis-induced aspiration pneumonia and bacteremia in an elderly man: a first case report and literature review
Source: BMC Infect Dis. 2023 May 4;23:285. doi: 10.1186/s12879-023-08269-x (PMC10157996; doi:10.1186/s12879-023-08269-x)
Supplement: Supplementary file 2 — Additional file 2: Table S1. Susceptibility results of a case described by Yu et al., 2020. [file 12879_2023_8269_MOESM2_ESM.docx]

Table S1 Susceptibility results of a case described by Yu et al., 2020

| Antibiotic | Antibiogram result | MIC* |
| --- | --- | --- |
| Colistin | Resistant | 8 |
| Amikacin | Susceptible | ≦2 |
| Aztreonam | Susceptible | ≦1 |
| Ceftazidime | Susceptible | ≦0.12 |
| Ciprofloxacin | Susceptible | ≦0.25 |
| Cefepime | Susceptible | ≦0.12 |
| Imipenem | Susceptible | ≦0.25 |
| Levofloxacin | Susceptible | ≦0.12 |
| Minocycline | Susceptible | ≦1 |
| Tobramycin | Susceptible | ≦1 |
| Piperacillin/Tazobactam | Susceptible | ≦4 |
| Meropenem | Susceptible | ≦0.25 |
| Ticarcillin/Clavulanic Acid | Susceptible | ≦8 |
| Trimethoprim/Sulfamethoxazole | Susceptible | ≦20 |
| Tigecycline | Susceptible | ≦0.5 |
| Cefoperazpne/Sulbactam | Susceptible | ≦8 |
| Doxycycline | Susceptible | ≦0.5 |

*MIC: minimal inhibitory concentration [μg/L]
