# Supplementary material for: Vogesella urethralis-induced aspiration pneumonia and bacteremia in an elderly man: a first case report and literature review
Source: BMC Infect Dis. 2023 May 4;23:285. doi: 10.1186/s12879-023-08269-x (PMC10157996; doi:10.1186/s12879-023-08269-x)
Supplement: Supplementary file 3 — Additional file 3: Table S2. Susceptibility results of a case described by Lan et al., 2020. [file 12879_2023_8269_MOESM3_ESM.docx]

Table S2 Susceptibility results of a case described by Lan et al., 2020

| Antibiotic | Antibiogram result* |
| --- | --- |
| Penicillin G | Susceptible |
| Ampicillin | Susceptible |
| Chloramphenicol | Susceptible |
| Gentamicin | Susceptible |
| Kanamycin | Susceptible |
| Nalidixic acid | Susceptible |
| Novobiocin | Susceptible |
| Rifampicin | Susceptible |
| Streptomycin | Susceptible |
| Tetracycline | Susceptible |
| Ciprofloxiacin | Susceptible |
| Levofloxacin | Susceptible |
| Erythromycin | Susceptible |
| Clindamycin | Resistant |
| Polymyxin B | Susceptible |
| Sulfamethoxazole/Trimethoprim | Susceptible |

*The susceptibility results were interpreted with reference to *Neisseriaceca* species as listed in CLSI M100-S27.
